# A Prospective Study of the Age at Menarche in North Indian Girls, Its Association With the Tanner Stage, and the Secular Trend

**DOI:** 10.7759/cureus.45383

**Published:** 2023-09-16

**Authors:** Aishwarya Bajpai, Utkarsh Bansal, Richa Rathoria, Ekansh Rathoria, Vijay Singh, Girjesh K Singh, Ravindra Ahuja

**Affiliations:** 1 Pediatrics, Hind Institute of Medical Sciences, Barabanki, IND; 2 Obstetrics and Gynecology, Uma Nath Singh Autonomous State Medical College, Jaunpur, IND; 3 Pediatrics, Uma Nath Singh Autonomous State Medical College, Jaunpur, IND

**Keywords:** menstruation, early menarche, adolescence, tanner stage, thelarche, menarcheal age

## Abstract

Background

Menarche is an important event in a female's reproductive health. However, the age at menarche is variable and has implications on the future health of the girl. The purpose of this study was to estimate the mean age at menarche of North Indian adolescent girls and its association with the Tanner stage and to study the trend of menarcheal age in India in the 21st century.

Materials & methods

A longitudinal descriptive study was conducted on 470 healthy girls aged nine to 16 years in expectant menarche (Tanner stage II). They were followed for six months to check for the attainment of menarche. Those who achieved menarche were grouped in Group I and the rest in Group II. The data were analyzed using the independent t-test.

Results

Menarche was achieved by 263 girls in the six-month follow-up period. The mean (SD) age of menarche was 13.13 (1.23) years. Group I girls were mostly in Tanner stage IV and above. Group II girls had a mean (SD) age of 11.53 (1.1) years and were mostly in Tanner stage III or below. The estimated decline in the age of menarche in the 21st century was about 0.41 years per decade.

Conclusion

The girls who achieved menarche had a significantly higher age and Tanner stage of sexual maturity than girls who did not achieve menarche in the study period. Tanner stage is a better measure to estimate the pubertal onset than age. The studies on the age of menarche in the current century reveal that the declining trend in the menarcheal age is continuing in India.

## Introduction

Menarche is the first period of a woman's menstruation, an event that represents reproductive potential and the passage from childhood to womanhood [1]. The average age for menarche is 12 years worldwide, which has declined in recent years [1]. Several studies have found that the average age at menarche in most developed nations has declined from 17 years in 1840 to around 12 years in 2000 [1-3].

A secular declining trend in the menarcheal age over the last two centuries has been demonstrated globally and Indian studies have proved the same [4-7]. The mean menarcheal age has declined from 16.50 years to 12.43 years over the last 40 years [4]. This gradual decline in the age of menarche is known to be associated with several types of mental and physical health issues in girls [4]. A systematic literature review of 112 studies from 1972 to 2019 concluded that the alteration in the age of menarche can have effects on the mental health, fertility, cardiovascular health, and bone health of women [1]. Early menarche is linked to obesity, unsafe sexual behavior, metabolic syndrome, and breast cancer [2,3]. Delay in menarche, on the other hand, raises the risk of depression, cardiometabolic problems, irregular menstrual cycles, and reduced peak bone density [2,3].

Menarche may be affected by genetics, ethnicity, climate changes, nutrition, physical activity, geographical region, urban or rural living conditions, well-being status, psychological variables, body mass, size of family, family income, parental education, parental control, loss of guardians, child sexual maltreatment, physical stress, and passive smoking [1,3,8].

A study based on the Indian Human Development Survey revealed that the age of menarche declined to 13.62 years in women born in 1985-1989, from 13.83 years in those born in 1955-1964 [4]. Indian studies regarding the age of attainment of menarche are mostly retrospective and reveal a range of 11.8 to 13.86 years in various parts of the country [4-10]. We planned the present study to estimate the age of menarche in North Indian girls prospectively and the association of the age at menarche with the Tanner stage. We also intended to study the trend of the age at menarche in the 21st century in India by reviewing the available studies.

## Materials and methods

This longitudinal descriptive study was conducted among adolescent girls aged between nine and 16 years with expectant menarche living in the Barabanki district of Uttar Pradesh in North India from December 2021 to December 2022. The study was approved by the Institutional Ethics Committee, Hind Institute of Medical Sciences on October 21, 2021 (HIMS/IRB/2021-22/3950).

We included adolescent girls aged nine to 16 years, who had not achieved menarche, who had attained thelarche (glandular breast tissue development), with sexual maturity rating (SMR) II or more according to the Tanner staging [11], and Indian ancestry; while excluding those with any known chronic illness (diabetes, hypertension, bronchial asthma, thyroid disorder, epilepsy), and on long-term medication (>two months) (Table 1).

**Table 1:** Tanner staging in females Adapted from: Marshall WA, Tanner JM. Variations in pattern of pubertal changes in girls. Arch Dis Child. 1969 Jun;44(235):291-303 [11].

Stage	Breast growth	Pubic hair growth
I	Pre-pubertal	No terminal hair
II	Breast bud appears (thelarche)	Sparse and straight hair seen along the labia
III	Breast enlargement generalized (extends beyond the areola)	Pubic hair increase and become pigmented, curly, and coarse
IV	The nipple and areola over the breast tissue form a second mound	Increase in the number of hair that spreads over the entire mons pubis
V	Mature adult-type breasts, areola recedes, and nipple projects	Pubic hair adult type in triangular shape area with spreading over to the medial thighs

**Figure 1:**
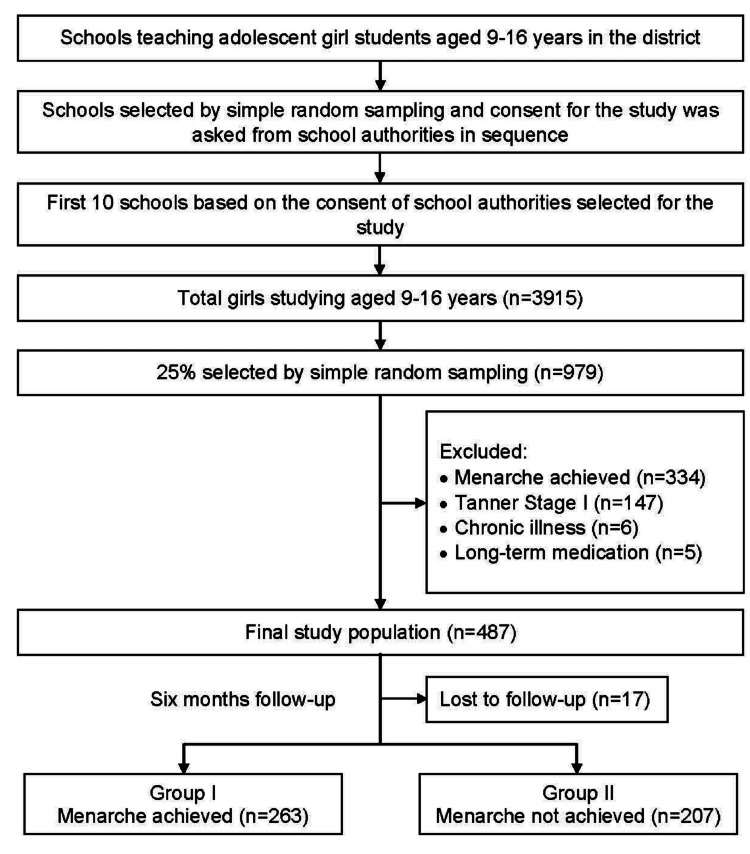
Flowchart illustrating the selection process of the study participants

Taking the total population data of Barabanki as 32,60,699 from the 2011 Census of India, with a male-to-female ratio of Barabanki as 1:0.91, among which 12.1% were adolescents, the sample size was calculated as 470 using OpenEpi software (at absolute precision of 5%, the confidence level of 97%, a design effect of 1, and hypothesizing that 50% of the study population will achieve menarche) [12]. A multistage stratified random sampling technique was used. A list of all the schools where adolescent girls were studying between the ages of nine and 16 years in the district was made (n = 417). In the second stage, we selected schools by simple random sampling and communicated with the authorities of the school, explained the objectives of the study, and asked for their consent to conduct the study. If they denied it, we approached the second school on the random list and sought consent. This approach was followed till we got consent from 10 schools. In the third stage, we asked for a list of adolescent girls from the age group of nine to 16 years, and 25% of the girls were selected by simple random sampling and shortlisted for inclusion in the study (Figure 1).

Data were collected on a structured questionnaire after obtaining informed consent from parents and assent from the study participants. The study participants were shown pictorial Tanner staging for sexual maturity and asked to identify to which stage they belong. Those who identified themselves in Tanner stage II or above were clinically examined by a female pediatrician to validate the pubertal staging of the breast. This helped to distinguish between glandular breast tissue and fat tissue [11]. Those in Tanner stage II or more were enquired if they were suffering from any chronic illness, were on any long-term medication, and had achieved menarche; and if any of the response was positive they were excluded from the study. The baseline anthropometric measures of the study subjects were done using standard methods, which included height in meters (m) using a stadiometer, and weight in kilograms (kg) using an electronic weighing scale. The body mass index (BMI) was calculated as weight divided by height squared. Then the participants were followed up telephonically over six months to check for the attainment of menarche. The schools were revisited twice, after three and six months of the initial visit to physically verify the girls who had achieved menarche. Those who attained menarche were grouped in Group I, and those not attaining menarche in Group II.

**Figure 2:**
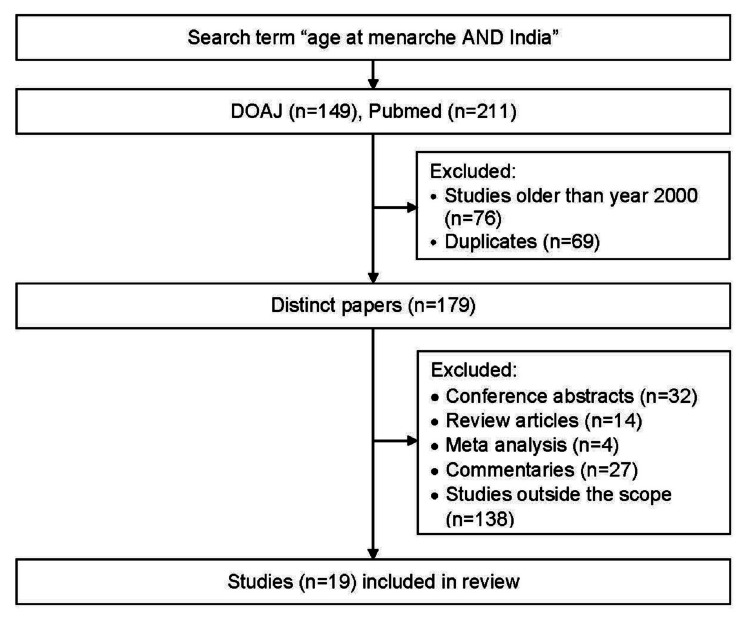
Flowchart illustrating the selection process of the studies on the age of menarche DOAJ: Directory of Open Access Journals.

We searched for relevant literature articles about the age of menarche using two databases: PubMed, and Directory of Open Access Journals (DOAJ). The search was done for studies published from the year 2000 onwards, using the keywords “age at menarche AND India.” The resulting studies were manually reviewed and the duplicates were removed. Only human studies and studies involving healthy girls were included. Conference abstracts, review articles, and commentaries were removed. The remaining studies were reviewed, arranged chronologically, and analyzed for the trend of menarcheal age (Figure 2).

Data analysis was performed using IBM SPSS Statistics version 26.0 (IBM Corp., Armonk, NY). The normality of distribution was checked using the skewness and kurtosis calculation, showing normal distribution when values were between −1.00 and 1.00. The independent t-test was used to compare the mean between both the groups concerning the age and Tanner staging. A p-value less than 0.05 was considered significant.

## Results

The age of the girls enrolled in the study ranged from 10 to 15 years with a median age of 12 years. The mean (SD) age of the study population was 12.42 (1.40) years (Table 2).

**Table 2:** Distribution of study population according to age (n = 470)

Age (in years)	Number of girls (n)	Percentage (%)
10	45	9.6
11	83	17.7
12	121	25.7
13	106	22.5
14	79	16.8
15	36	7.7

**Figure 3:**
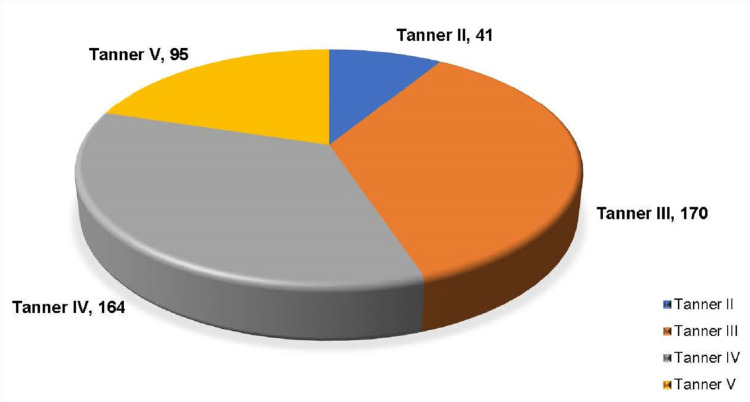
Distribution of study population according to Tanner stage (n = 470)

The majority of the girls were in Tanner stage III (170, 36.2%) (Figure 3).

During the study period (six months from the initial evaluation), a total of 56% of girls attained menarche. The mean (SD) age of menarche was 13.13 (1.23) years. There were 263 (56%) girls in Group I who achieved menarche while 207 (44%) girls did not achieve menarche. The mean (SD) BMI of girls achieving menarche was 18.43 (3.39) kg/m^2^ while that of girls not achieving menarche was 16.12 (2.39) kg/m^2^.

**Figure 4:**
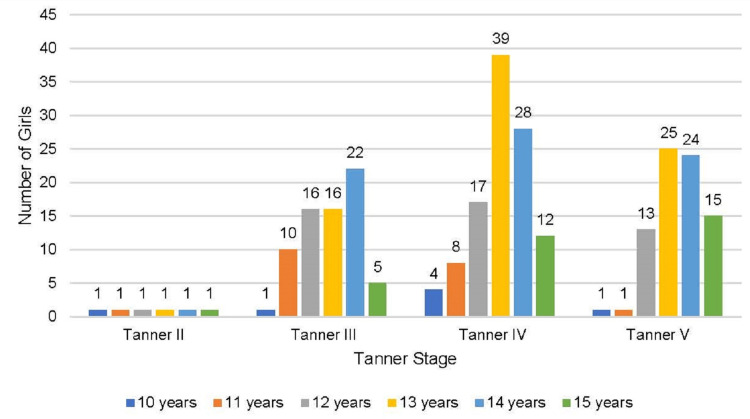
Distribution of girls achieving menarche (Group 1) according to Tanner staging and age (N = 263)

**Figure 5:**
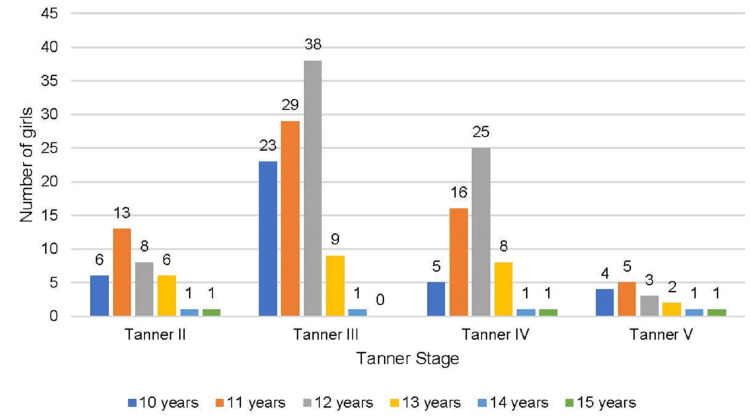
Distribution of girls not achieving menarche (Group II) according to Tanner staging and age (N = 207)

The majority of the girls achieving menarche were aged ≥12 years (71.9%) and had a Tanner stage above stage III (71.1%) whereas the majority of the girls failing to achieve menarche were aged <12 years (84.5%) and had a Tanner stage below or equal to stage III (65.2%) (Figures 4, 5).

The mean age and Tanner stage were significantly higher in those achieving menarche as compared to those not achieving menarche (Table 3).

**Table 3:** Comparison of age and Tanner staging between Group I (N=263) and Group II (N = 207) * Significant p-value < 0.05.

Variables	Group I	Group II	Independent t-test	
	Mean (SD)	Mean (SD)	t-value	p-value*
Age	13.13 (1.23)	11.53 (1.1)	14.78209	<0.00001
Tanner stage	3.99 (0.81)	3.26 (0.83)	9.61919	<0.00001

**Figure 6:**
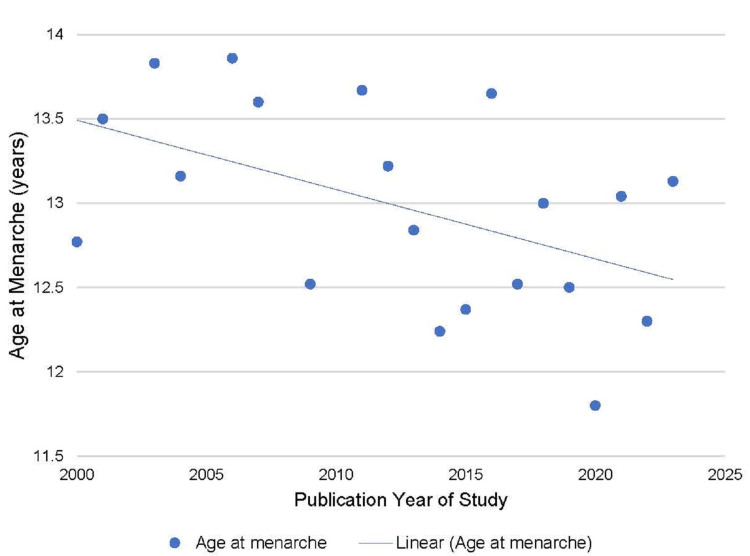
Trend in the age of menarche estimated in studies done in India in the 21st century

There were 19 Indian studies found on the age of menarche that met the review criteria. The mean age of menarche observed by different studies done in India from the year 2000 onwards was plotted against the study year and the trend line was observed [5-10,13-25]. The trend indicated that there is a consistent lowering in the age of menarche. The estimated decline was about 0.41 years per decade (Figure 6).

## Discussion

Adolescence is an important transitional period from childhood to adulthood in which changes occur first concerning physical maturation, reproductive capabilities, and then in psychological and social maturity as well [26]. Menarche is a significant milestone in human biological development and is frequently seen as a fertility indicator [3]. Menarche can occur at any age in a girl, although it is most common between the ages of nine and 16 years [27]. The present study was planned to study the average age of menarche and its association with the Tanner stage. The trend of menarcheal age in the current century reported by Indian researchers was also studied.

Cross-sectional studies in Northern India reported the mean (SD) age at menarche as 13.5 years in 380 respondents [13], 13.86 (0.01) years in 100 girls aged nine to 15 years [10], 13.6 years in 251 girls aged 10-19 years [14], 12.84 (1.4) years in 847 girls aged 10-19 years [15], 12.98 (0.77) years in 120 girls aged 13-18 years [16], and 12.4 years in 2010 school girls aged six to 17 years [17]. Our study participants had a mean (SD) age at menarche of 13.13 (1.23) years.

Studies from Western India documented the mean (SD) menarcheal age as 12.77 (1.05) years in 366 girls aged nine to 16 years [5], 13.16 (1.10) years in 168 girls aged 10-19 years [18], 12.62 (1.05) years in 742 girls aged nine to 16 years [6], and 12.5 (1.01) years in 100 girls aged 10-15 years old [19].

The mean (SD) age of menarche was 13.83 (0.87) years in 250 females [20], 12.5 (1.42) years in 100 females [7], and 13 (1.1) years in 536 females aged 10-19 years [21] in South Indian studies.

In Eastern India, the mean (SD) age of menarche was observed to be 12.35 (0.02) years in 453 girls aged 10-16 years [22], 12.24 (0.73) years in 541 girls aged 13-18 years [9], 11.8 (1.2) years in 2195 females aged seven to 21 years [23], and 12.45 years in 463 girls aged nine to 16 years old [8].

The studies from central India have estimated the mean (SD) age of menarche as 13.67 (0.8) years in 1100 adolescent girls aged 10-19 years old [24] and 13.2 (1.24) years in 492 girls aged 11 to 18 years [25].

It may be noted that most of these studies were retrospective, unlike the present study, which was prospective and included adolescent girls with expectant menarche instead of a cross-sectional study that focused on menarche status at the time of enrolment. The prospective method to assess the menarcheal age is considered better than the recall method [4]. Originally, we intended to include girls aged nine to 16 years of age in the study as it comes under the normal age of menarche. However, we did not find any girl aged nine years in Tanner stage II and all girls aged 16 years had already achieved menarche, hence girls aged nine and 16 years were automatically excluded from the study.

Tanner staging ought to be utilized instead of chronological age to determine pubertal development [28]. Menarche usually occurs after thelarche (breast budding) within two to three years, at Tanner stage IV breast development, and is uncommon before Tanner stage III [27]. In the present study, 263 (56%) girls achieved menarche (Group I) while 207 (44%) girls did not achieve menarche (Group II) during the six-month follow-up period. The mean age and the mean Tanner staging of girls in Group I were significantly higher as compared to those in Group II (Table 3). On reviewing the contemporary literature, we did not find any other study evaluating the relationship between the onset of menarche with age and the Tanner stage.

An analysis based on the Indian Human Development Survey has noted a secular decline in age at menarche among Indian women [4]. The study on age at menarche in low- and middle-income countries (LMICs) established a lowering from 14.66 to 12.86 years for the 1932 and 2002 cohorts [29]. The review of Indian studies done in the 21st century revealed a similar secular trend in the age of menarche (Figure 6) [5-10,13-25]. The studies were from all parts of the country and the differences observed in the age of menarche can be attributed to environmental, racial, and genetic factors. It has been documented in previous studies that the age of menarche in urban girls is lower than that of rural girls [4]. Our study population involved girls mainly of a rural background, which explains the higher mean age of menarche noted in our study. The improvement in socioeconomic conditions and health in the 20th century is considered the main cause of the early-onset menarche seen in girls of developed countries, and a similar trend is seen in the developing world also [1,3]. The age of menarche seems to be settled now in the industrialized world [3]. Glandular breast tissue development (thelarche) is a sensitive indicator of onset of puberty and age at thelarche has shown a similar declining trend as age at menarche [30].

The strength of the present study is the prospective study design, the inclusion of the Tanner stage to find out girls with expectant menarche, and regular follow-up till attainment of menarche. This is a novel concept, as most previous studies have been cross-sectional or retrospective.

The study is limited by the sample size and geographical area. We were not able to do a longer follow-up, which could have increased the sample size.

## Conclusions

The present study attempted to study the average age at menarche in North Indian adolescent females prospectively. Menarche was achieved by 56% of the participants during the study follow-up period. The mean (SD) menarcheal age was 13.13 (1.23) years. The girls achieving menarche as compared to those not achieving menarche were significantly older and in higher Tanner stages. Further studies on a larger sample size are recommended to elaborate on and substantiate these relationships. The onset of puberty is related to the Tanner stage and thelarche can be utilized to identify girls in the expectant menarche. The secular trend of the age of menarche is noticed in the studies done in India in the current century.
